# Association between adherence to the Mediterranean diet with cardiometabolic risk factors: a cross-sectional study on PERSIAN cohort study in Fasa

**DOI:** 10.1038/s41598-023-41935-3

**Published:** 2023-09-08

**Authors:** Milad Bagheri, Mehran Nouri, Reza Homayounfar, Masoumeh Akhlaghi

**Affiliations:** 1 Department of Community Nutrition, School of Nutrition and Food Sciences, Shiraz University of Medical Sciences, Razi Blvd, Shiraz, Iran; 2 Students’ Research Committee, Shiraz University of Medical Sciences, Shiraz, Iran; 3 Non-Communicable Diseases Research Center, Fasa University of Medical Sciences, Fasa, Iran; 4 Faculty of Nutrition Sciences and Food Technology, National Nutrition and Food Technology Research Institute, Shahid Beheshti University of Medical Sciences, Tehran, Iran

**Keywords:** Biomarkers, Diseases, Health care, Medical research, Risk factors, Signs and symptoms

## Abstract

The relationship between Mediterranean diet and obesity-related markers is a matter of debate. We investigated the association between adherence to the Mediterranean diet and anthropometric indices, body composition, and cardiometabolic risk factors in Iranian population. The cross-sectional study was performed on data of 3386 participants from Fasa PERSIAN cohort study. The Mediterranean diet score (MDS) was calculated based on consumption of 11 food groups (unrefined cereals, potatoes, fruits, vegetables, legumes, fish, red meat, poultry, dairy, olive oil, and alcoholic beverages). The association between MDS and cardiometabolic risk factors was examined by linear regression analysis. MDS was inversely associated with waist circumference (β** = ** − 1.11; P = 0.033), waist-to-hip ratio (β =  − 0.007; P = 0.011), waist-to-height ratio (β =  − 0.009; P = 0.015), fasting glucose (β =  − 3.59; P = 0.001), and HDL-cholesterol (β =  − 0.96; P = 0.031) in unadjusted model. After adjusting for energy intake, the associations of MDS with markers of abdominal obesity and HDL-cholesterol disappeared. In fully adjusted model, MDS showed inverse relationships with waist-to-hip ratio (β =  − 0.005; P = 0.037) and fasting glucose (β =  − 2.71; P = 0.013). In conclusion, MDS showed an inverse relationship with fasting glucose and waist-to-hip ratio. Since energy intake increased along with increasing MDS, adherence to the Mediterranean diet may associate with lower abdominal obesity and better glycemic control if an energy-controlled Mediterranean diet is used.

## Introduction

The prevalence of obesity has been increasing during the last decades. According to the World Health Organization (WHO) report, the worldwide prevalence of obesity has tripled since 1975^1^, and is projected to reach 18% in men and more than 21% in women by 2025^2^. The rate of obesity is estimated 18–23% in studies conducted in Iranian population^3–5^. Obesity is associated with pathological conditions such as cardiovascular disease, type 2 diabetes, dyslipidemia, and some kinds of cancer^6^, leading to 2.8 million annual deaths worldwide^1^. Therefore, obesity prevention greatly reduces the financial burden imposed on healthcare systems.

WHO defines obesity as an abnormal or excessive accumulation of fat, which may impair health^1^. Body mass index (BMI) is an indicator that is commonly used to diagnose obesity, but measuring body composition is a better tool for diagnosing obesity because it gives information on body fat mass while BMI makes no distinction between adipose tissue and lean mass^7^. Fat distribution in the body is also an important factor in determining the risk of chronic metabolic diseases^8^. Waist circumference, waist to hip, and waist to height ratios are indicators of abdominal obesity that can be used along with BMI to give more information about obesity^9^.

The Mediterranean diet is a well-known healthy diet which contains high quantities of vegetables, fruits, whole grains, legumes, and nuts, has olive oil as the primary source of fat, and is moderate in fish and poultry, low to moderate in dairy and eggs, limited in red meat and sweets, and permits moderate amounts of red wine^10^. The health effects of the Mediterranean diet are much debated today. Although there is relative consensus on its beneficial effects on cardiovascular health^10^, studies on the effect of this diet on fat mass and other obesity markers have shown conflicting results. Some studies have indicated an inverse association between the Mediterranean diet and obesity-related markers^11,12^, while in others the relationship has not been significant^13–15^. Due to inconsistencies in the results of previous investigations and the lack of studies conducted in Iran, we investigated the association between adherence to the Mediterranean diet and anthropometric indices, body composition, and cardiovascular risk factors in participants of a cohort study in Iran. The results provided new suggestions for improving the effectiveness of the Mediterranean diet against obesity and related cardiometabolic diseases.

## Methods

### Study design and ethics

This cross-sectional study was performed on the baseline data of the PERSIAN cohort study of Fasa city in Shiraz province of Iran. PERSIAN cohort study is a prospective national study that was started in 2014 and is currently underway in 18 regions of Iran. The cohort study was approved by the Ethics Committee of Fasa University of Medical Sciences (IR.FUMS.REC.1395.177) at that time and the study was conducted in accordance with the Helsinki declaration and Iranian national guidelines for ethics in research. The protocol of the PERSIAN cohort study has been published before^16^. Before data collection, the participants were informed about the study process and signed a consent form.

### Participants

All adults aged 35 to 70 years living in rural regions of Fasa were invited to participate in the study. There were no specific inclusion criteria and all individuals living in rural geographical areas of Fasa were included. However, individuals with physical or psychological disabilities who were unable to perform the measurements or respond the questions were excluded^16^. Overall, 10,127 individuals were included in the original cohort study; of which 4,658 had body composition data and were considered for the present study.

### General information

Demographic information including age, sex, marital status, educational level, and smoking was collected through personal interviews. Physical activity was determined with a 20-item questionnaire designed to measure the activity of Iranians in rural areas, as described before^17^. The time of each activity (in hours) per day was multiplied by the MET-value of that activity and the physical activity of each person was recorded as MET/day.

### Anthropometric and body composition measurements

Weight was measured by a digital scale with an accuracy of 0.1 kg. Height was measured using a stadiometer with an accuracy of 0.1 cm. BMI was calculated by dividing weight in kg by the square of height in meters. Waist circumference was measured using a non-elastic tape with an accuracy of 0.1 cm in the narrowest part between the lowest rib and the head of the iliac crest. With the same tape, hip circumference was estimated at the greatest protrusion of the buttocks. Waist to hip and waist to height ratios were calculated. Fat mass, fat-free mass, and trunk fat were assessed using a bioelectrical impedance analysis (BIA) device (Tanita BC-418, Tanita Company, Tokyo, Japan). Fat mass index and fat-free mass index were calculated by dividing fat mass and fat-free mass by the square of height in meters, respectively.

### Cardiovascular risk factors

To assess metabolic factors, venous blood samples were taken after 10–14 h fasting, and the level of triglycerides, total cholesterol, HDL cholesterol, LDL cholesterol, and fasting glucose was assessed using an automatic analyzer (Mindray Medical International, Shenzhen, China). Systolic and diastolic blood pressure was measured by a nurse, after 5 min rest, twice at intervals of 15 min in a sitting position with a standard mercury sphygmomanometer, and the average of the two measurements was recorded.

### Dietary assessment

Dietary intakes were questioned by a 125-item food frequency questionnaire (FFQ), which was designed based on the Willett-type questionnaire^18^. The participants were asked to report the type, frequency, and amount of food items on a daily, weekly, monthly, or annual basis during the last year^19^. Dietary intakes were analyzed using Nutritionist IV software 2 (Hearst Corp., San Bruno, CA) and daily intake of energy and nutrients was determined.

### Mediterranean diet score

The adherence to the Mediterranean diet was determined using the Mediterranean diet score (MDS) tool^20^. To calculate MDS, the daily intake of 11 food groups (unrefined cereals, potatoes, fruits, vegetables, legumes, fish, red meat and its products, poultry, high-fat dairy, olive oil, and alcoholic beverages) was scored from 0 to 5^20^. The score of 0 was assigned for no consumption of non-refined cereals, potatoes, fruits, vegetables, legumes, fish, and olive oil, consuming > 700 ml/day alcoholic beverages (> 84 g ethanol/day) or no alcohol consumption, and > 5 servings of red meat and its products, poultry, and full fat dairy products. The score of 5 was given for consuming > 18 servings/month of non-refined cereals, potatoes, fruits, vegetables, legumes, and fish, daily consumption of olive oil, ingestion of < 300 ml/day alcoholic beverages (< 36 g ethanol/day), and no consumption of red meat, poultry, and full fat dairy products. The consumption of the foods between these levels was scored accordingly. Scores of individual items were summed to obtain the MDS. The MDS ranges from 0 to 55, with the higher scores indicating greater adherence to the Mediterranean diet^20^.

### Statistical analysis

For data analysis, SPSS software version 20 (IBM Corp., Endicott, New York, United States) and STATA version 14 (STATA Corp., College Station, Texas, United States) were used. Participants were divided into tertiles based on MDS, and general information, physical activity, energy intake, anthropometric characteristics, body composition parameters, and cardiovascular risk factors were expressed in MDS tertiles and compared with Chi-square (for categorical variables) or one-way analysis of variance (ANOVA) (for quantitative variables) test. The association between the mentioned parameters and MDS was investigated using linear regression in three models of crude (model I), adjusted based on energy intake (model II), and adjusted additionally for age, sex, marital status, educational level, smoking, and physical activity (model III). In all statistical tests, P < 0.05 was considered statistically significant.

### Ethical approval and consent to participate

The results presented herein were extracted from the thesis written by Mr. Milad Bagheri. The study was approved by Shiraz University of Medical Sciences, Shiraz, Iran (approval number 22359). All methods performed in the current study were in accordance with the Declaration of Helsinki.

## Results

### General information of participant

**Figure 1:**
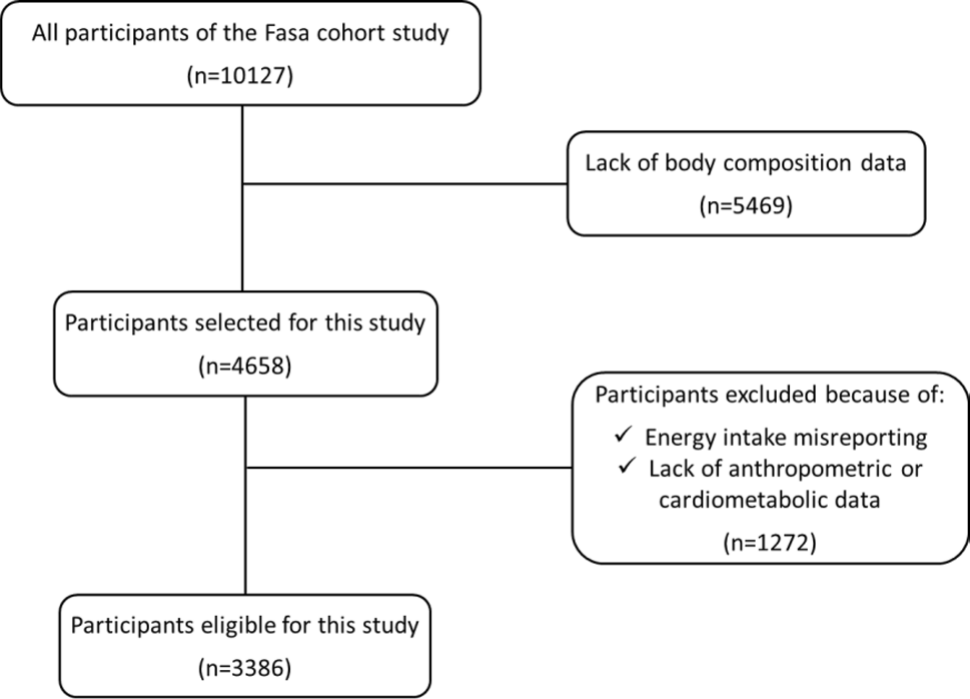
Participant inclusion flow diagram.

Data of 4658 participants were initially considered, but 1272 were excluded due to misreporting dietary items (i.e. energy intake outside the range of 800–4200 kcal/day for men and 600–3600 kcal/day for women) or missing anthropometric or cardiometabolic data. Finally, 3386 participants were included in the study: 1436 men (42.4%) and 1950 women (57.6%). Figure 1 demonstrates the flowchart of the participant selection. General information, anthropometric characteristics, body composition, and cardiovascular risk factors of the participants in MDS tertiles are summarized in Table 1. There was no significant difference in age, sex, marital status, and educational level between MDS tertiles. Energy intake increased across MDS tertiles (P < 0.001). Among the cardiometabolic variables, only waist to hip ratio, fasting glucose, and HDL cholesterol differed between MDS tertiles: participants with higher MDS had lower values of these parameters (P < 0.05).

**Table 1:** Descriptive characteristics of participants across MDS tertiles.

Variable	MDS			
	T_1_ (n = 1475)	T_2_ (n = 985)	T_3_ (n = 926)	P value^a^
Age, years	48.7 ± 9.6	48.1 ± 9.4	48.1 ± 9.4	0.20
Sex, n (%), male	608 (41.2)	419 (42.5)	409 (44.2)	0.36
Marital status, n (%)				
Single	61 (4.1)	32 (3.2)	37 (4.0)	0.07
Married	1280 (86.8)	894 (90.8)	824 (89.0)	
Widow, divorced	134 (9.1)	59 (5.5)	65 (7.0)	
Education, n (%)				
School	1439 (97.6)	950 (96.4)	892 (96.3)	0.15
Smoking history, n (%)				
Yes	361 (24.5)	267 (27.1)	247 (26.7)	0.27
Physical activity (MET/day)	41.1 ± 10.1	41.6 ± 10.2	41.4 ± 11.0	0.44
Energy intake (kJ/day)	10,840 ± 2931	11,486 ± 2844	11,723 ± 2861	** < 0.001**
Height (cm)	160.8 ± 8.8	160.8 ± 9.0	161.3 ± 9.0	0.34
Weight (kg)	66.0 ± 12.7	65.9 ± 12.7	65.9 ± 13.2	0.98
Body mass index (kg/m^2^)	25.6 ± 4.9	25.6 ± 4.8	25.4 ± 4.9	0.54
Waist circumference (cm)	93.0 ± 12.0	92.6 ± 11.9	91.9 ± 12.2	0.09
Hip circumference (cm)	99.7 ± 8.8	99.3 ± 8.4	99.3 ± 9.1	0.45
Waist to hip ratio	0.93 ± 0.07	0.93 ± 0.07	0.92 ± 0.07	**0.02**
Waist to height ratio	0.58 ± 0.09	0.58 ± 0.09	0.57 ± 0.09	0.05
Fat percentage	28.3 ± 10.1	27.8 ± 9.9	27.5 ± 10.1	0.12
Fat mass (kg)	19.4 ± 9.1	19.0 ± 8.7	18.9 ± 9.3	0.29
Fat-free mass (kg)	47.0 ± 8.2	47.4 ± 8.5	47.5 ± 8.4	0.35
Systolic blood pressure (mmHg)	109.3 ± 18.6	109.0 ± 18.5	108.7 ± 17.4	0.72
Diastolic blood pressure (mmHg)	72.6 ± 12.4	72.3 ± 11.8	72.6 ± 11.7	0.80
Fasting glucose (mg/dl)	94.0 ± 31.2	89.8 ± 21.2	90.4 ± 24.0	** < 0.001**
Triglyceride (mg/dl)	125.8 ± 78.1	126.9 ± 75.2	127.9 ± 75.0	0.80
Total cholesterol (mg/dl)	185.8 ± 39.3	182.5 ± 38.4	183.7 ± 37.9	0.11
HDL cholesterol (mg/dl)	48.0 ± 10.4	46.9 ± 10.4	47.1 ± 10.2	**0.01**
LDL cholesterol (mg/dl)	112.6 ± 31.6	110.2 ± 31.3	111.0 ± 31.6	0.17

Values are means ± SD for continuous and number and percentage for categorical variables.

^a^P values were determined with one-way ANOVA for continuous and Chi-square test for categorical variables.

Significant values are in bold.

### Anthropometric indices and MDS

Examining the association of anthropometric indices with MDS tertiles showed that with increasing MDS, waist circumference (β =  − 1.11; 95% CI − 2.10, − 0.13), waist to hip ratio (β =  − 0.007; 95% CI − 0.012, − 0.002), and waist to height ratio (β =  − 0.009; 95% CI − 0.016, − 0.002) significantly decreased (Table 2). However, after adjustment for energy intake, the association between MDS and the anthropometric indices was no longer significant. After addition of age, sex, marital status, educational level, smoking, and physical activity in the model, only the inverse relationship between MDS and waist to hip ratio becomes significant although the regression coefficient was quite small (β =  − 0.005; 95% CI − 0.010, − 0.0003).

**Table 2:** Association of anthropometric parameters with MDS tertiles.

Variable	MDS			
	T_1_ (n = 1475)	T_2_ (n = 985)	T_3_ (n = 926)	P value^a^
Weight				
Model I^b^	Ref.	− 0.08 (− 1.10, 0.96)	− 0.09 (− 1.14, 0.97)	0.871
Model II^c^	Ref.	− 0.34 (− 1.38, 0.69)	− 0.46 (− 1.52, 0.60)	0.433
Model III^d^	Ref.	− 0.09 (− 1.08, 0.90)	− 0.45 (− 1.46, 0.56)	0.392
BMI				
Model I	Ref.	− 0.05 (− 0.45, 0.34)	− 0.22 (− 0.63, 0.18)	0.274
Model II	Ref.	0.09 (− 0.30, 0.48)	− 0.04 (− 0.44, 0.36)	0.858
Model III	Ref.	0.048 (− 0.31, 0.40)	− 0.17 (− 0.54, 0.20)	0.379
Waist circumference				
Model I	Ref.	− 0.41(− 1.38, 0.55)	− 1.11 (− 2.10, − 0.13)	0.033
Model II	Ref.	− 0.26 (− 0.99, 0.93)	− 0.58 (− 1.57, 0.40)	0.251
Model III	Ref.	− 1.01 (− 0.99, 0.79)	− 0.87 (− 1.78, 0.03)	0.060
Waist to hip ratio				
Model I	Ref.	− 0.001 (− 0.006, 0.004)	− 0.007 (− 0.012, − 0.002)	0.011
Model II	Ref.	0.0014 (− 0.004, 0.007)	− 0.004 (− 0.009, 0.001)	0.136
Model III	Ref.	0.0012 (− 0.004, 0.006)	− 0.005 (− 0.010, − 0.0003)	0.037
Waist to height ratio				
Model I	Ref.	− 0.003 (− 0.009, 0.004)	− 0.009 (− 0.016, − 0.002)	0.015
Model II	Ref.	0.003 (− 0.004, 0.009)	− 0.002 (− 0.009, 0.005)	0.588
Model III	Ref.	0.0003 (− 0.005, 0.006)	− 0.005 (− 0.010, 0.0004)	0.072

^a^The values are linear regression coefficient and 95% confidence interval. Tertile 1 was considered as the reference.

^b^P value for tertile 3 in comparison to tertile 1.

^c^Crude.

^d^Adjusted for energy intake.

^e^Additionally adjusted for age, sex, marital status, educational level, smoking, and physical activity.

### Body composition and MDS

Body fat percentage (β =  − 0.82; 95% CI − 1.64, 0.01; P = 0.052) and fat mass index to fat-free mass index ratio (β =  − 0.016; 95% CI − 0.032, 0.0002; P = 0.052) showed a trend for a significant inverse relationship with MDS tertiles in the crude model (Table 3). However, in the controlled models none of body composition parameters had a significant relationship with MDS tertiles.

**Table 3:** Association of body composition parameters with MDS tertiles.

Variable	MDS			
	T_1_ (n = 1475)	T_2_ (n = 985)	T_3_ (n = 926)	P value^a^
Fat percentage				
Model I^b^	Ref.	− 0.59 (− 1.40, 0.22)	− 0.82 (− 1.64, 0.01)	0.052
Model II^c^	Ref.	0.140 (− 0.63, 0.90)	0.17 (− 0.60, 0.96)	0.659
Model III^d^	Ref.	− 0.26 (− 0.80, 0.30)	− 0.37 (− 0.90, 0.18)	0.194
Fat mass				
Model I	Ref.	− 0.47 (− 1.20, 0.26)	− 0.51 (1.25, 0.23)	0.180
Model II	Ref.	− 0.06 (− 0.77, 0.66)	0.05 (− 0.68, 0.79)	0.887
Model III	Ref.	− 0.27 (− 0.88, 0.35)	− 0.31 (− 0.94, 0.32)	0.332
Fat-free mass				
Model I	Ref.	0.35 (− 0.32, 1.03)	0.47 (− 0.21, 1.16)	0.181
Model II	Ref.	− 0.34 (− 0.97, 0.29)	− 0.48 (− 1.12, 0.16)	0.144
Model III	Ref.	0.09 (− 0.37, 0.54)	− 0.13 (− 0.59, 0.34)	0.601
Trunk fat to total body fat				
Model I	Ref.	− 0.48 (− 1.28, 0.32)	0.12 (− 0.69, 0.94)	0.306
Model II	Ref.	− 1.25 (− 2.00, − 0.49)	− 0.92 (− 1.69, − 0.15)	0.022
Model III	Ref.	− 0.63 (− 1.10, 0.15)	− 0.34 (− 0.83, 0.15)	0.179
Fat mass index				
Model I	Ref.	− 0.19 (− 0.5, 0.11)	− 0.26 (− 0.57, 0.05)	0.101
Model II	Ref.	0.03 (− 0.26, 0.33)	0.04 (− 0.26, 0.34)	0.777
Model III	Ref.	− 0.09 (− 0.32, 0.15)	− 0.13 (− 0.38, 0.11)	0.283
Fat-free mass index				
Model I	Ref.	0.13 (− 0.04, 0.29)	0.06 (0.10, 0.23)	0.474
Model II	Ref.	0.04 (− 0.12, 0.20)	− 0.06 (− 0.22, 0.10)	0.475
Model III	Ref.	0.10 (− 0.05, 0.25)	− 0.02 (− 0.18, 0.13)	0.770
Fat mass index/fat-free mass index				
Model I	Ref.	− 0.013 (− 0.03, 0.003)	− 0.016 (− 0.032, 0.0002)	0.052
Model II	Ref.	0.001 (− 0.014, 0.016)	0.003 (− 0.012, 0.018)	0.684
Model III	Ref.	− 0.007 (− 0.017, 0.004)	− 0.007 (− 0.018, 0.004)	0.215

^a^The values are linear regression coefficient and 95% confidence interval. Tertile 1 was considered as the reference.

^b^P value for tertile 3 in comparison to tertile 1.

^c^Crude.

^d^Adjusted for energy intake.

^e^Additionally adjusted for age, sex, marital status, educational level, smoking and physical activity.

### Cardiometabolic risk factors and MDS

Examining the association of cardiometabolic risk factors and MDS revealed that with increasing MDS, fasting glucose (β =  − 3.59; 95% CI − 5.78, − 1.40) and HDL cholesterol (β =  − 0.96; 95% CI − 1.81, − 0.11) significantly decreased, but after eliminating the effect of confounding variables, the relationship between MDS and HDL cholesterol became insignificant (P > 0.05) (Table 4). Other cardiometabolic risk factors did not show a significant association with MDS in any of the regression models.

**Table 4:** Association of cardiovascular risk factors with MDS tertiles.

Variable	MDS			
	T_1_ (n = 1475)	T_2_ (n = 985)	T_3_ (n = 926)	P value^a^
Fasting glucose				
Model I^b^	Ref.	− 4.24 (− 6.40, − 2.09)	− 3.59 (− 5.78, − 1.40)	0.001
Model II^c^	Ref.	− 3.60 (− 5.76, 1.46)	− 2.72 (− 4.92, − 0.52)	0.015
Model III^d^	Ref.	− 3.43 (− 5.53, − 1.35)	− 2.71 (− 4.85, − 0.58)	0.013
Triglyceride				
Model I	Ref.	1.12 (− 5.05, 7.28)	2.13 (− 4.15, 8.41)	0.507
Model II	Ref.	0.68 (− 5.51, 6.87)	1.53 (− 4.80, 7.86)	0.643
Model III	Ref.	1.07 (− 5.10, 7.23)	1.62 (− 4.69, 7.93)	0.609
Total cholesterol				
Model I	Ref.	− 3.27 (− 6.38, − 0.15)	− 2.11 (− 5.29, 1.07)	0.193
Model II	Ref.	− 2.87 (− 6.00, 0.26)	− 1.56 (− 4.76, 1.63)	0.338
Model III	Ref.	− 3.08 (− 6.16, − 0.01)	− 1.93 (− 5.08, 1.21)	0.232
LDL cholesterol				
Model I	Ref.	− 2.37 (− 4.91, 0.17)	− 1.57 (− 4.16, 1.02)	0.238
Model II	Ref.	− 2.20 (− 4.75, 0.36)	− 1.33 (− 3.94, 1.28)	0.321
Model III	Ref.	− 2.28 (− 4.81, 0.25)	− 1.52 (− 4.10, 1.07)	0.259
HDL cholesterol				
Model I	Ref.	− 1.12 (− 1.95, − 0.28)	− 0.96 (− 1.81, − 0.11)	0.031
Model II	Ref.	− 0.81 (− 1.64, 0.25)	− 0.54 (− 1.39, 0.31)	0.226
Model III	Ref.	− 1.02 (− 1.81, − 0.23)	− 0.74 (− 1.55, 0.07)	0.068
Systolic blood pressure				
Model I	Ref.	− 0.38 (− 1.85, 1.09)	− 0.60 (− 2.1, 0.90)	0.434
Model II	Ref.	− − 0.01 (− 1.48, 1.46)	− 0.01 (− 1.60, 1.40)	0.909
Model III	Ref.	0.24 (− 1.14, 1.61)	− 0.01 (− 1.41, 1.39)	0.995
Diastolic blood pressure				
Model I	Ref.	− 0.33 (− 1.3, 0.64)	− 0.08 (− 1.07, 0.91)	0.882
Model II	Ref.	− 0.25 (− 1.23, 0.72)	0.02 (− 0.98, 1.02)	0.968
Model III	Ref.	− 0.13 (− 1.08, 0.83)	0.07 (− 0.91, 1.04)	0.893

The values are linear regression coefficient and 95% confidence interval. Tertile 1 was considered as the reference.

^a^P value for tertile 3 in comparison to tertile 1.

^b^Crude.

^c^Adjusted for energy intake.

^d^Additionally adjusted for age, sex, marital status, educational level, smoking, and physical activity.

### Dietary intakes and MDS

Dietary macro- and micro-nutrient intakes generally increased across MDS tertiles, except for fat, trans-fats, cholesterol, sucrose, and vitamin B_12_ that did not show a trend and saturated fats and mono-unsaturated fats that decreased across the tertiles (Table 5).

**Table 5:** Nutrient intakes of participants across the MDS tertiles.

Variable	MDS			
	T1 (N = 1475)	T2 (N = 985)	T3 (N = 926)	P value^a^
Energy (kcal/day)	2591 ± 700	2745 ± 680	2802 ± 684	< 0.001
Carbohydrate (g/day)	439 ± 127	476 ± 123	490 ± 124	< 0.001
Protein (g/day)	77.1 ± 22.9	83.2 ± 23.4	84 ± 23.5	< 0.001
Fat (g/day)	62.1 ± 23.8	60.3 ± 22.6	60.9 ± 24.2	0.15
Trans-fats (g/day)	0.17 ± 0.16	0.18 ± 0.18	0.19 ± 0.17	0.12
Saturated fats (g/day)	24.1 ± 11.9	22.9 ± 11.4	22.5 ± 12.0	< 0.01
Mono-unsaturated fats (g/day)	19.0 ± 8.6	17.9 ± 7.9	18.2 ± 8.7	< 0.01
Poly-unsaturated fats (g/day)	8.41 ± 3.86	8.56 ± 4.02	9.32 ± 4.16	< 0.001
Cholesterol (mg/day)	237 ± 121	234 ± 126	231 ± 135	0.558
Total fiber (g/day)	23.0 ± 8.5	26.0 ± 8.9	28.7 ± 9.5	< 0.001
Fructose (g/day)	22.9 ± 13.9	24.6 ± 13.9	27.3 ± 15.8	< 0.001
Sucrose (g/day)	53.2 ± 41.7	53.6 ± 38.7	57.0 ± 44.0	0.067
Vitamin A (IU/day)	11,688 ± 9520	12,686 ± 10,670	13,950 ± 11,021	< 0.001
Vitamin K (µg/day)	187 ± 189	206 ± 162	227 ± 183	< 0.001
Vitamin E (mg/day)	5.77 ± 3.02	6.06 ± 2.87	6.76 ± 2.97	< 0.001
Thiamin (B_1_) (mg/day)	1.72 ± 0.62	1.80 ± 0.66	1.89 ± 0.77	< 0.001
Vitamin B_6_ (mg/day)	1.29 ± 0.47	1.38 ± 0.52	1.51 ± 0.54	< 0.001
Vitamin B_9_ (µg/day)	328 ± 171	362 ± 185	400 ± 203	< 0.001
Vitamin B_12_ (µg/day)	2.47 ± 2.09	2.38 ± 1.95	2.49 ± 2.5	0.477
Vitamin C (mg/day)	113 ± 71	124 ± 73	140 ± 81	< 0.001
Calcium (mg/day)	452 ± 208	494 ± 249	518 ± 257	< 0.001
Magnesium (mg/day)	238 ± 89	259 ± 101	284 ± 104	< 0.001
Sodium (mg/day)	3184 ± 1130	3433 ± 1107	3470 ± 1186	< 0.001
Potassium (mg/day)	2846 ± 1182	3099 ± 1292	3425 ± 1346	< 0.001
Selenium (mg/day)	43.3 ± 20.3	46.3 ± 22.3	50.1 ± 25.8	< 0.001

Values are means ± SD.

^a^P values were determined with one-way ANOVA.

## Discussion

### Main findings

In this cross-sectional study that was performed on 3386 adults aged 35–70 years participating in the Fasa PERSIAN cohort study, we investigated the association between adherence to the Mediterranean diet and anthropometric indices, body composition, and cardiometabolic risk factors. The results of this study showed that abdominal obesity markers including waist circumference, waist to hip, and waist to height ratios had a weak but significant inverse association with MDS tertiles. However, after control of potential confounders only waist to hip ratio remained statistically significant. Among biochemical cardiometabolic risk factors, fasting blood glucose was the only parameter that showed an inverse association with MDS tertiles after adjustment for confounding variables.

### MDS and anthropometric indices

In this investigation, MDS showed a weak inverse relationship with waist circumference and waist to hip and waist to height ratios, as indicators of abdominal obesity, but it was not significantly related to markers of general obesity such as weight and BMI. However, after adjusting for energy intake, no association between parameters of abdominal obesity and MDS was observed, suggesting that a combination of high energy intake and good adherence to the Mediterranean diet was associated with decreased abdominal obesity. By eliminating the effect of other confounders (model III), the inverse relationship between MDS and waist to hip ratio, but not waist circumference and waist to height ratio, became significant again, although the association was still weak. Further analysis showed that sex and smoking had the greatest impact on the relationship of MDS with waist to hip ratio. In fact, women and non-smokers had higher waist to hip ratio compared to men and smokers, and thus after addition of sex and smoking in the model, the relationship between MDS and waist to hip ratio became statistically significant.

### MDS and body composition

There was no significant association between body composition variables and MDS. This finding was not unpredictable as no significant relationship was found between obesity indices (i.e. weight and BMI) and MDS, and among indicators of abdominal obesity, only waist to hip ratio showed a weak relationship with MDS. However, trunk to body fat ratio showed a significant inverse relationship with MDS after adjusting for energy intake, suggesting that greater adherence to the Mediterranean diet without increased energy intake could be associated with lower trunk to body fat ratio. Again, in the fully adjusted model, the significance of the association disappeared.

In line with our results, Boghossian et al. reported lower trunk to leg fat ratio in reproductive aged women with higher scores of the Mediterranean diet^21^. However, in contrast to us, they also found the association of the MDS with all adiposity measures except waist to hip ratio. In the same direction, Prieto-González et al. reported correlations between better adherence to the Mediterranean diet and lower BMI and body fat in college males and a very weak correlation with waist to hip ratio^22^. Consistent with our findings, waist-related variables such as waist circumference and waist to height ratio have been the only anthropometric measures which inversely associated with adherence to the Mediterranean diet in some investigations^23–25^ but there are also studies in which no association was found with measures of general or abdominal obesity or body fat^13^. There is much controversy regarding the relationship of the Mediterranean diet and obesity-related markers and more studies and perhaps meta-analyses are needed to clarify the issue.

### MDS and cardiometabolic risk factors

In the crude regression model, there was an inverse association between MDS and fasting glucose and HDL cholesterol, but after removing the effect of the confounders, only the inverse association between fasting glucose and MDS remained statistically significant. Further analysis showed that energy intake had the greatest confounding effect on the relationship between HDL cholesterol and MDS. The energy intake increased along with increasing MDS score (r = 0.16, P < 0.001). Besides, energy intake inversely correlated with HDL level (r =  − 0.14, P < 0.001). Thus, by controlling energy intake in the model the relationship between MDS and HDL cholesterol has been weakened and lost its significance.

Similar to other cardiometabolic risk factors, the relationship of blood glucose and the Mediterranean dietary pattern is controversial and both cases of an inverse^22,26,27^ or no^26,28,29^ relationship have been reported. The same scenario exists for HDL cholesterol as there are reports on both positive (i.e. beneficial)^24,30^ and no^31^ relationship with the Mediterranean diet.

The inverse association of the Mediterranean diet and blood glucose may be due to inverse association that was found between the Mediterranean diet and waist to hip ratio. Individuals who more adhered to the Mediterranean diet had significantly lower waist to hip ratio and lower blood glucose. The Mediterranean diet also has components which could help in better blood glucose control. These include, but not limited to, dietary fibers which slows down glucose absorption rate, antioxidants that protect beta cells against oxidative stress, and oleic acid present in olive oil which increases adiponectin and prevents insulin resistance^32^. Moreover, many metabolic abnormalities are speculated to relate to mitochondria dysfunction as a result of nutrient deficiencies. The Mediterranean diet contains nutritionally valuable foods such as fruits and vegetables, nuts, whole grains, legumes, fish, and olive oil^33^. Nutrients that are provided by these foods improve mitochondrial function and thus may help in prevention of obesity-related cardiometabolic diseases.

### Reasons of controversies

The heterogeneity in findings between studies could be due to differences in participant characteristics, study design, or methodological limitations. For instance, differences in the rate of physical activity can affect obesity outcomes. In countries with low levels of physical activity, the adherence to the Mediterranean dietary pattern may be only weakly related to measures of obesity^25^. Moreover, in countries where following a Mediterranean diet style is not a general habit of people, the adherence to the Mediterranean diet by individuals is probably accompanied with selection of other healthy lifestyles such as enough physical activity and good sleep health, factors that also contribute to weight control and obesity prevention. But this may not be the case in Sothern Europe countries where people habitually follow a Mediterranean-type diet and ignore other recommendations for a healthy lifestyle. The possibility of a reverse causality in the relationship of the Mediterranean diet and general obesity should also be considered, as obese individuals are more inclined to select healthy diets.

### Strengths and limitations

The present study had strengths and limitations. The large sample size used generally increases the accuracy of the findings and reduces the margin of error, but it may also amplify differences and suggest results that are not clinically important. The cross-sectional design of the study did not allow to draw cause-and-effect relationships, and prospective or interventional studies are needed to prove that. The intake of some components of the Mediterranean diet is generally low among Iranians. For example, olive oil is one of the main components of the Mediterranean diet, but in Iran due to limited cultivation of olives and the high price, the intake of olives and olive oil is much less than Mediterranean countries. In addition, in Iran due to legal and religious restrictions, consumption of alcoholic beverages is very low or underreported. These dietary restrictions may impede Iranians from using a real Mediterranean diet and thus may reduce generalizability of the results.

## Conclusion

Overall, results of this study showed an inverse relationship between adherence to the Mediterranean diet and fasting blood glucose and waist to hip ratio in fully adjusted model, although the magnitude for waist to hip ratio was small and may not be of clinical value. Also, waist circumference had an inverse relationship and body fat percentage showed a trend for an inverse relationship with MDS but after adjustment for energy intake both relationships lost their statistical significance. These results suggest that although greater adherence to the Mediterranean diet may be associated with higher energy intake as a result of increased food consumption, the higher energy intake may not be associated with increased risk of overweight/obesity or other cardiometabolic risk factors. Instead, higher intake of the Mediterranean diet foods may be associated with lower risk of fasting blood glucose, abdominal obesity, and body fat percentage. Due to the cross-sectional design, cause and effect relationships cannot be proven based on results presented herein and controlled interventional investigations are needed to make better conclusions in this matter.

## Data Availability

Data are available upon request from the corresponding author.
